# Chronic venous ulcers: a review on treatment with fibrin sealant and prognostic advances using proteomic strategies

**DOI:** 10.1590/1678-9199-JVATITD-2019-0101

**Published:** 2020-06-22

**Authors:** Luciana Patricia Fernandes Abbade, Rui Seabra Ferreira, Lucilene Delazari dos Santos, Benedito Barraviera

**Affiliations:** 1 Department of Infectology, Dermatology, Imaging Diagnosis and Radiotherapy, Botucatu Medical School (FMB), São Paulo State University (UNESP), Botucatu, SP, Brazil.; 2 Graduate Program in Clinical Research, Botucatu Medical School (FMB), São Paulo State University (UNESP), Botucatu, SP, Brazil.; 3 Graduate Program in Tropical Diseases, Botucatu Medical School (FMB), São Paulo State University (UNESP), Botucatu, SP, Brazil.; 4 Center for the Study of Venoms and Venomous Animals (CEVAP), São Paulo State University (UNESP), Botucatu, SP, Brazil.

**Keywords:** Biological dressings, Proteomics, Venous ulcers, Fibrin tissue adhesive, Biopolymers

## Abstract

Venous ulcers are the main causes of chronic lower-limb ulcers. The healing difficulties encourage the research and development of new products in order to achieve better therapeutic results. Fibrin sealant is one of these alternatives. Besides being a validated scaffold and drug delivery system, it possesses excellent healing properties. This review covered the last 25 years of the literature and showed that the fibrin sealant is used in various clinical situations to promote the healing of different types of ulcers, especially chronic ones. These are mostly venous in origin and usually does not respond to conventional treatment. Commercially, only the homologous fibrin sealants obtained from human blood are available, which are highly efficient but very expensive. The heterologous fibrin sealant is a non-commercial experimental low-cost product and easily produced due to the abundance of raw material. The phase I/II clinical trial is already completed and showed that the product is safe and promisingly efficacious for the treatment of chronic venous ulcers. In addition, clinical proteomic strategies to assess disease prognosis have been increasingly used. By analyzing liquid samples from the wounds through proteomic strategies, it is possible to predict before treatment which ulcers will evolve favorably and which ones will be difficult to heal. This prognosis is only possible by evaluating the expression of isolated proteins in exudates and analysis using label-free strategies for shotgun. Multicentric clinical trials will be required to evaluate the efficacy of fibrin sealant to treat chronic ulcers, as well as to validate the proteomic strategies to assess prognosis.

## Background

Venous ulcers (VUs) are responsible for about 70% of chronic ulcers of the lower limbs, which occur in the regions below the knees and do not heal within a six-week period. They affect a large portion of the adult population, cause significant economic and social impact [1], and diminish the quality of life [2]. 

In the United States about 2 million persons are affected annually [3] resulting in an annual estimated cost for treatment between 1.9 and 2.5 billion dollars. In the United Kingdom, 1.3% of the health budget is invested in treatment of VUs [4]. In Brazil, Castro and Silva [5] reported that chronic venous disease, in its diverse clinical presentations as VUs, ranked fourteenth among causes of workplace absences. 

After healing, recurrence is about 30% in the first year and 78% after two years. This occurs when compressive therapy or surgical correction of venous disease is not adopted correctly [6]. 

VUs are, therefore, a serious public health problem for western countries, in part due to their augmented prevalence, long healing time, frequent recurrence and elevated treatment costs, impacting ultimately the life quality of the patient. 

## Topical Treatments

Topical treatment of VUs is fundamental, given that it promotes ulcer bed preparation and accelerates the healing process [7]. 

According to Falanga [8] it is ineffective to utilize sophisticated high-cost products in poorly prepared wounds. To obtain an efficacious result, an ulcer must have at least a well vascularized bed, a minimal quantity of bacteria and little or no inflammatory exudate. Preparing the ulcer bed involves the control of the diverse steps, namely the acronym TIME that means Tissue, Inflammation/infection, Moisture imbalance and Epithelial edge advancement [9]. It describes four aspects of wound bed preparation that need to be systematically addressed for wound healing to take place.

Research on topical products for VU treatment has been under development for decades. This has contributed to a significant increase in the availability of new products. Approximately 2,500 items are currently on the market for acute and chronic ulcers, from the simplest coverage and antiseptic solutions to the most complex dressings, which actively interfere in the various phases of the healing process [10].

Effective dressings are those that not only maintain the ideal environment for the healing process but also have a functional effect [11,12]. Currently, some products possess these characteristics, but they are considered high tech, which often renders them unviable due to elevated costs and prolonged time of treatment.

Among the diverse substances utilized as alternatives is fibrin sealant. Besides its important role in hemostasis, its fibrin resulting from the polymerization of fibrinogen, together with the matrix of proteins, acts in the healing process to promote angiogenesis, collagen synthesis, as well as wound contraction and reepithelization [13-15].

## Fibrin Sealant

Fibrin sealant is a biocompatible product, mainly of topical use, whose performance is based on fibrin polymerization resulting from the reaction between fibrinogen and the final products of the blood coagulation cascade [16]. It is composed basically of human fibrinogen and thrombin, therefore it is usually called “homologous fibrin sealant”. In the presence of small quantities of calcium and factor XIII, the thrombin converts the fibrinogen in insoluble fibrin, polymerizing and forming a stable network. Some presentations of fibrin sealant also have aprotinin, an antifibrinolytic agent whose objective is to retard clot fibrinolysis.

Research into the development of this biomaterial began during World War II in order to develop a product with adhesive and hemostatic capacity for use in surgical procedures. At that time, the goal was to develop a substance that besides being adhesive and hemostatic, would provide stable adhesion among tissues, favoring the repair process [17]. 

At first, human thrombin and fibrinogen were employed in a product generally denominated “fibrin glue”, whose components were mixed during the procedure and at the application site. Around 1944, autologous plasma enriched with autologous fibrinogen, i.e., a person's own blood products, was utilized. At that point, these experiments were not successful. Again in 1970, the proposal of the “fibrin glue” was reevaluated and then renamed fibrin sealant. On that occasion, the basic principles for producing fibrinogen-rich cryoprecipitate and factor XIII, among other coagulation factors, were already known. Furthermore, the purification of thrombin from the blood of large animals was also standardized. The purpose of this new sealant would be to mix human fibrinogen-rich cryoprecipitate with bovine thrombin plus calcium chloride during the desired procedure. This proposal was commercialized over the years by pharmaceutical laboratories with relative success. The disadvantage of the homologous fibrin sealant is that it uses products derived from a pool of human blood donors, which could lead to the possibility of transmission of infectious diseases such as hepatitis, acquired immunodeficiency syndrome, parvovirus B19, syphilis, Chagas disease, among others [18, 19]. Thus, in 1978, The U.S. Food and Drug Administration (FDA) suspended the license for the commercialization of this product in U.S. territory [20, 21]. 

Recently, with the improvement of laboratory diagnoses of infectious diseases, the homologous fibrin sealant has been reapproved in the United States by the FDA, and has become available again on the market. Approval was obtained in 1998 for the liquid presentation, and in 2010 for the patch form. Its use was indicated for the following purposes: hemostatic, sealant and adhesive [17]. The hemostatic function is mainly indicated for surgical procedures; the sealant property refers to a seal that impedes the leaking of liquid or gas from a structure in, for example, intestinal, vascular and cardiac surgeries, whereas the adhesive function promotes the gluing of such structures as grafts and flaps of skin. Thus, the fibrin sealant currently available on the market is indicated mainly for cardiovascular, pulmonary, abdominal, neurological and other surgical procedures. Furthermore, it is indicated for hemostasis in skin and mucosae and for the treatment of ulcers that present difficulty in healing [17]. Table 1 displays the products commercialized in the U.S. 

**Table 1.** Fibrin sealants available in the U.S. and approved by the FDA.

Function	Origin	Commercial name
Hemostatic and sealant	Pool of human plasma	Tisseel^®^, Baxter Healthcare Corp., Westlake Village, CA
Hemostatic	Pool of human plasma	Evicel^®^, Ethicon/J&J, Somerville, NJ
Hemostatic	Individual plasma, bovine collagen and thrombin	Vitagel^®^, Orthovita/Stryker, Malvern, PA
Hemostatic	Pool of human plasma and equine collagen	Tachosil^®^, Baxter Healthcare Corp., Westlake Village, CA
Hemostatic	Pool of human plasma and regenerated oxidized cellulose	Evarrest^®^, Ethicon/J&J, Somerville, NJ
Adhesive	Pool of human plasma	Artiss^®^, Baxter Healthcare Corp., Westlake Village, CA

Source: adapted from Spotnitz [17].

Among the products listed in Table 1 and approved for use in Brazil by the Brazilian Health Regulatory Agency (ANVISA) are Tisseel^®^, Evicel^®^ and Tachosil^®^. Besides these, Denmark has commercialized the product Beriplast^®^, which is also approved by ANVISA for use in Brazil. This product contains, in addition to human products, aprotinin, an antifibrinolytic agent of animal origin.

## Clinical Studies of Fibrin Sealant in Chronic Ulcers

The benefits described for utilizing fibrin sealant in chronic ulcers are related to hemostasis and to greater adherence of the graft to the ulcer bed. Besides its adherence function, fibrin sealant is an excellent biological scaffold. Its characteristic of adhering to the ulcer bed, for at least four days, makes it an excellent drug-delivery structure for incorporating and releasing cells, growth factors and even antibiotics [22]. This biopolymer is completely degraded in a period of ten days.

The present study also describes in chronological order the main clinical studies in which fibrin sealant was employed in the treatment of chronic ulcers.

In 1988, Hunyadi et al. [23] applied a fibrin sealant (Beriplast^®^) associated or not with autologous keratinocytes to treat 20 patients with VU and five with third-degree burns. In 16 of the 20 patients treated with fibrin sealant associated with autologous keratinocytes, there was complete healing within 14 to 21 days. On the other hand, fibrin sealant without keratinocytes did not significantly accelerate the reepithelization process. 

Dahlstom et al. [24], in 1992, utilized autologous fibrin sealant to seal skin grafts in seven patients with chronic ulcers on the lower limbs. These ulcers were divided into two equal parts, and the sealant was employed to attach of split thickness skin grafts in half of the ulcer. The biopsies performed on the seventh day after surgery showed that grafts were more stable in sealant-fixed areas. The biopsies obtained on the 21^st^ postoperative day did not differ significantly between the two treatments.

Calabrò et al. [25], in 1995, performed a non-randomized study applying fibrin sealant directly on the bed of chronic lower-limb ulcers of 80 patients. Fifty-four patients were treated with the traditional method and the remaining 26 with fibrin sealant. They concluded that weekly fibrin-sealant treatment, besides reducing the pain, had a favorable impact, reducing the healing time. 

In 2000, the group of Siedler et al. [26] reported one case in which commercial homologous fibrin sealant was used together with a culture of allogenic keratinocytes (from a human donor) to treat one lower-limb chronic ulcer of arteriovenous etiology refractory to other treatments. After 10 weeks of weekly application there was improvement of granulation tissue, permitting cutaneous autograft as the final treatment. 

Kopp et al. [27] treated 11 patients in 2004 with chronic lower-limb ulcers that were refractory to diverse treatments with autologous keratinocytes and commercial fibrin sealant (Tisseel^®^). The patients received between one and three applications. Ten patients (90.9%) presented total healing. In 2005, Johnsen et al. [28] treated 60 patients with chronic lower-limb ulcers of diverse etiologies with a mix of autologous keratinocytes and fibrin sealant (BioSeed^®^-S, Germany). The healing rate was 55.8% after six weeks of treatment.

Vanscheidt et al. [29] in 2007 published a multicentered randomized clinical trial on VUs, in which they evaluated the effect of a combination of autologous keratinocytes with commercial fibrin sealant (BioSeed^®^-S, Germany). Forty-four (38.3%) of 116 patients, who received cell therapy along with three applications of fibrin sealant, presented complete healing of the target ulcer in the three-month period in comparison with 24 (22.4%) of the 109 patients who received only the standard treatment (p = 0.0106). This result demonstrated superior efficacy of the therapy of autologous keratinocytes associated with fibrin sealant.

In the next year, 2008, Hartmann et al. [30] monitored seven patients with VUs for six months, utilizing as treatment a culture of autologous keratinocytes mixed with commercial fibrin sealant (Tissucol^®^, Germany, an equivalent to Tisseel^®^ in the USA) in a single application. Complete healing was observed in four of the six ulcers after a mean duration of 14.5 weeks of observation. They concluded that the therapy with autologous keratinocytes suspended in fibrin is efficient in treating VUs.

In 2010, Chen et al. [31] described a new technique combining a gel of allogenic platelets from a single human donor, autologous skin graft and autologous fibrin sealant for treatment of chronic lower-limb ulcers that presented difficulty in healing. Fifteen patients with 17 chronic ulcers of various etiologies were treated. The interval between initial treatment and complete ulcer healing ranged from 3 weeks to 2 months. There was no recurrence after 3 to 18 months of follow-up. No adverse reactions were observed.

Dinato et al. [32], in 2012, described a technique for treatment of six chronic ulcers of diabetic origin using fibroblasts and keratinocytes from autologous skin cultured *ex vivo* and autologous fibrin sealant. Five patients received a single application and one received two applications. Complete healing was observed in five ulcers (83.3%) after 21 to 120 days of treatment. The patient with the largest ulcer showed partial improvement within 40 days. 

In 2012, Kirsner et al. [33] published a double-blind, controlled randomized clinical trial containing 228 patients with VUs divided into five groups, and monitored for 12 weeks. In the four intervention groups, neonatal dermal keratinocytes and fibroblasts were used every 7 or 14 days administered by spray pump on commercial fibrin sealant (Tisseel^®^) as well as a foam dressing composed of hydro-polymers. The control group received only the vehicle every 7 days, which consisted of a solution containing human fibrinogen. All five groups received four-layer compression bandage changed once a week. The primary outcome showed statistically significant greater reduction of wound area associated with active treatment in relation to the control group. The effects of a dose of 0.5 x 10⁶ cells/mL every 14 days were better when compared to the use of vehicle alone (15.98%, IC95% 5.56-26.41, p = 0.0028). The authors concluded that the VUs can be healed with a spray formulation of allogenic neonatal keratinocytes and fibroblasts dissolved in fibrin sealant. The ideal dose of cells applied for both fibroblasts and keratinocytes was 0.5 × 10⁶ cells per mL every 14 days. 

Asadi et al. [34] in 2014 published a new technique for the treatment of difficult-to-heal chronic ulcers. They used the combination of homologous platelets, homologous fibrin sealant and commercial collagen matrix in ten patients with aggressive and refractory ulcers. The combined therapy was applied every two days. There was complete healing in nine patients and the area was markedly reduced in one. There was no evidence of a local or systemic adverse event. 

In 2015, Velasco et al. [35] performed a cohort study containing 27 patients with spinal cord injury submitted to surgical treatment of pressure ulcers. Before surgical closure, commercial fibrin sealant (Tissucol^®^) was applied directly into the lesions. The costs and results obtained in this cohort were compared with those from a previous retrospective study containing 71 patients that were submitted only to conventional surgery. The sealant group presented lower rates of hematoma-seroma (3.7% vs. 33.8%; p < 0.05), lower mean drain volume (155 vs. 360 mL; p < 0.05) and significantly shorter hospitalization time than the historic group (40 days vs. 55 days; p < 0.05). They concluded that the application of commercial fibrin sealant during pressure ulcer surgery in patients with spinal cord injury demonstrated efficaciousness in reducing not only postoperative complications but also hospitalization time with consequent saving of financial resources.

The present review of more than 25 years demonstrates that fibrin sealant is used in a variety of clinical situations to promote healing of different types of ulcers, with major importance in chronic difficult-to-heal VUs and those that have not responded to conventional treatment. In most studies, autologous fibrin sealant or a homologous (commercial) one was used for fixation of grafts or as a scaffold for incorporation of cells, mainly keratinocytes. Ulcers presented improvement, with complete healing or reduction of their areas in most patients who used this therapy. However, only two clinical trials were randomized, with different treatments, impairing the obtainment of a good level of scientific evidence for this type of proposal. Therefore, it is fundamental that new studies are carried out, especially randomized clinical trials with a sample size sufficient to achieve clinical and statistical significance, in order to evidence the efficacy and safety of these treatments.

## Heterologous Fibrin Sealant

Despite all the precautions taken by manufacturers in the production of traditional sealants that utilize a pool of human plasma, the risk of transmission of new or even old viruses, as long as laboratory tests do not detect them, remains. Furthermore, the high production costs due to raw material scarcity make its routine use in the treatment of venous chronic ulcers unfeasible. The price for a single application of such commercial product ranges from 150 to 400 American dollars.

To circumvent these problems, since the 1990s the Center for the Study of Venoms and Venomous Animals (CEVAP) of UNESP has been researching to develop a new fibrin sealant. After several experiments, a heterologous fibrin sealant was standardized. It is constituted by a fibrinogen-rich cryoprecipitate extracted from the blood of *Bubalus bubalis* buffaloes and from a thrombin-like enzyme (serinoprotease) obtained from *Crotalus durissus terrificus* snake venom. The lack of human blood derivatives prevents the aforementioned transmission of infectious diseases [36, 37].

The polymerization of the heterologous fibrin sealant is based on the same principles as those of commercial sealants. The thrombin-like enzyme acts on the fibrinogen molecule, transforming it into monomers of fibrin that in the presence of calcium and other clotting factors present in the cryoprecipitate are polymerized to form a stable clot with adhesive, hemostatic and sealant effects [36, 37]. 

Throughout more than 20 years, preclinical studies on heterologous fibrin sealant have been performed. The acute cutaneous toxicity tests on rats and rabbits showed that the dose limit of 2,000 mg of fibrin sealant was not lethal to rats or rabbits, and did not produce systemic effects after 24 hours of cutaneous exposure. The genotoxicity potential tests (*Salmonella/*microsomal mutagenicity test) also did not present mutagenic activity in the five lineages tested up to the concentration of 200 µg/plate by the method of pre-incubation in the presence and absence of metabolic activation of the liver in rats. Thus, the test substance was approved and considered non-mutagenic. These results were requirements that were approved by the the Brazilian Health Regulatory Agency (ANVISA) to perform clinical trial phase I/II.

In addition to these studies, the heterologous fibrin sealant was tested experimentally on rodents [38, 39, 40, 41, 42, 43, 44] dogs [45, 46, 47, 48] and sheep [49, 50], as detailed in a recent systematic review by Buchaim et al [37].

The results of the toxicity and mutagenicity studies, together with preclinical studies on animals permitted the conclusion that the heterologous fibrin sealant was safe for human studies [51, 52, 53]. Furthermore, it was shown efficacious on account of its good performance in adhesion and hemostasis as well as in tissue repair of experimental animals.

**Figure 1.**
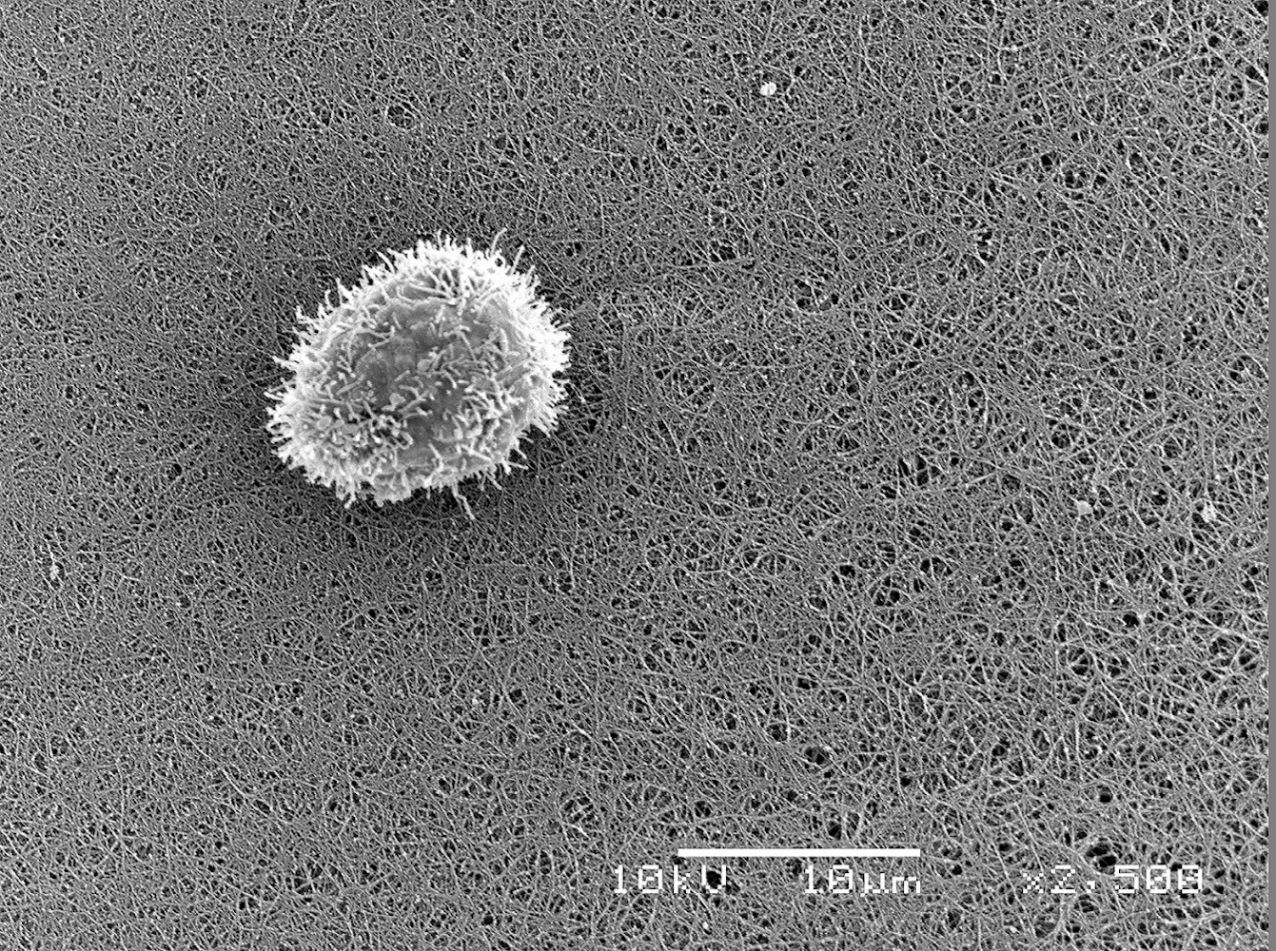
Scanning microscopy analysis of the heterologous fibrin sealant (HFS) (2,500x).

In 2018 Spejo et al. [54] used the heterologous fibrin sealant (HFS) in injured neurons in animal models showing the increase of the macrophage influx to the local lesion within 3 and 7 days. The HFS-treated group presented increased gene expression of M1 and M2 macrophage markers releasing anti-inflammatory and proinflammatory cytokines, as observed by qRT-PCR technics. Thus, it is possible to hypothesize that the heterologous fibrinolysis process can change the local environment generating a favorable predominantly proinflammatory state to start the healing process. Figure 1 shows a cell captured by the fibrin network formed by the HFS highlighting its drug-delivery system capacity and its ability to integrate with the environment in accordance with the previous results obtained by Orsi et al. [55]. 

At the beginning of the 21^st^ century, at the Botucatu Medical School (FMB) of São Paulo State University (UNESP), Brazil, a line of research was initiated on the application of heterologous fibrin sealant to VUs. This was based on the literature reporting healing properties of commercial sealant, namely the capacity to decrease infection and edema, control bleeding, increase pain threshold by protecting free nerve endings, moisturize the ulcer bed, and form granulation tissue [20, 21]. 

In 2009, Gatti et al. [56] treated for the first time 25 patients with VUs with heterologous fibrin sealant, and concluded that the product was promising, being cheaper than those available on the market, and presenting the following advantages: easy application, wound bed preparation tendency, and pain attenuation. The authors concluded that weekly application, for at least eight weeks, could improve the healing process.

Abbade et al. [57], in 2015, assessed ten patients with chronic VUs in a pilot study called Sealant Study I. This first project had not only trained the team, but had also prepared and standardized the product management and patient care for the Sealant Study II. The second phase was approved by the Brazilian Health Regulatory Agency (ANVISA) for future registration of HFS and distribution in the Brazilian public health system. The objectives of this second study were to evaluate the safety of the sealant in the VU healing process, as well as determining the best safe dose for covering the ulcers. 

**Figure 2.**
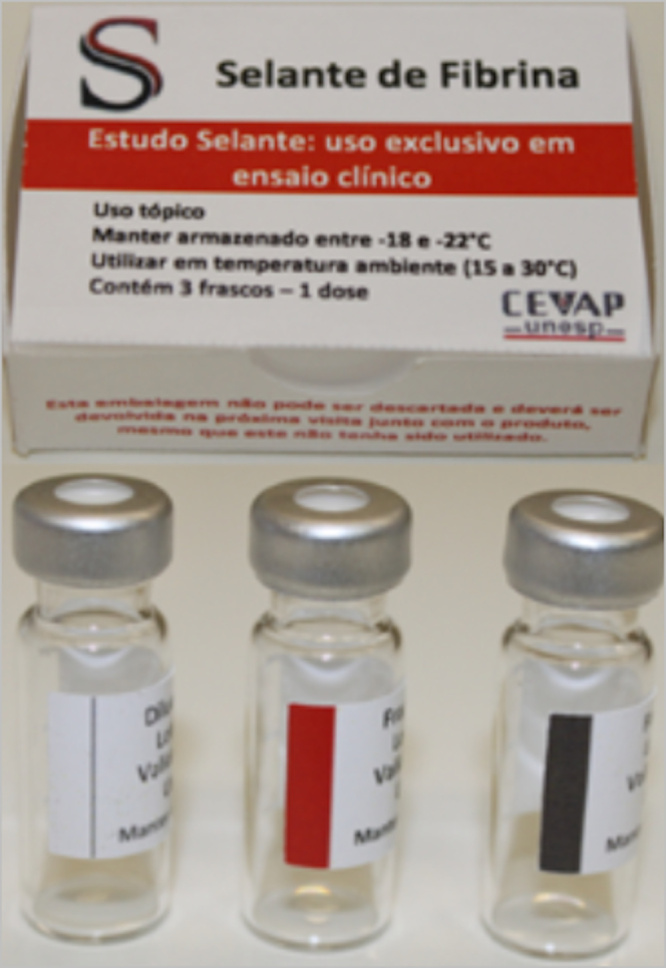
Package with three vials that constitutes the heterologous fibrin sealant. Diluent bottle (white stripe) containing 0.6 mL of calcium chloride; fraction 1 bottle (red stripe) containing 0.4 mL of serinoprotease from *Crotalus durissus terrificus* venom; fraction 2 bottle (black stripe) containing 1 mL of fibrinogen-rich cryoprecipitate and clotting factors extracted from buffaloes. This product was approved for utilization in clinical trials only.

**Figure 3.**
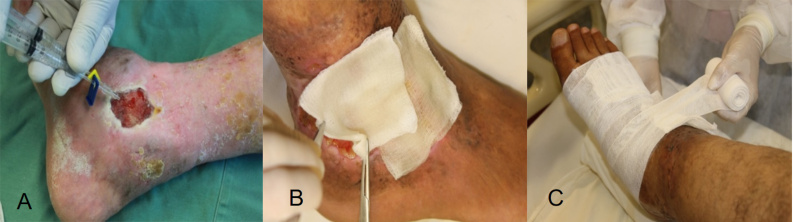
(A) Application of the product to an ulcer with fibrin polymerization to form a colorless gel in between 2 and 5 minutes. (B) Gauze soaked in essential fatty acids, in a quantity sufficient to cover the entire ulcerated surface. (C) Application of Unna’s boot dorsally from the foot up to just below the knee.

In Sealant Study I, ten participants with 18 ulcers were studied and treated for three months with direct weekly application of the sealant to the ulcer that was then covered with gauze soaked in essential fatty acids and subsequently compressed by an Unna’s boot (Figures 2 and 3). The total sealant dose utilized per patient varied between 6 and 22.8 with a mean of 12.8 doses. Each dose contained 2 mL of the final product. It was observed that the utilization of a dressing with the fibrin sealant showed, in relation to the primary outcomes, absence of severe adverse events, lack of systemic adverse events related to the product and no laboratory alterations with clinical significance. As secondary outcomes, 38.8% of the ulcers healed and 33.3% ulcers had reduction of areas with bed preparation, totaling a significant improvement in 72.1% of the cases. 

**Figure 4.**
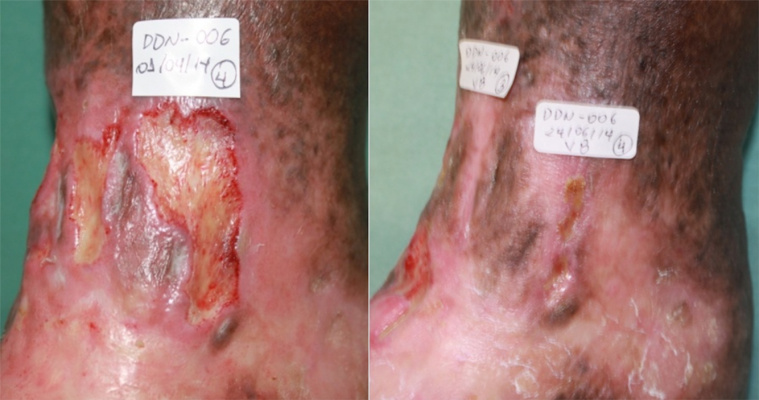
Female patient aged 66 years and carrier of four VUs for one year. Initial ulcerated area was 33.5 cm^2^, area at the final visit was 10.4 cm^2^ (two ulcers healed completely).

**Figure 5.**
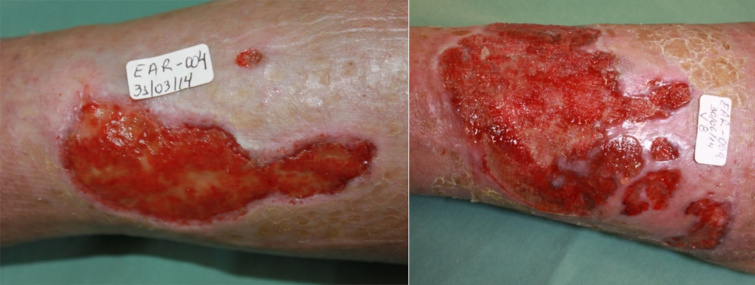
Female patient aged 76 years and carrier of one VU for three years. Initial ulcerated area was 25.4 cm^2^, area at the final visit was 52.0 cm^2^ (increasing from initial ulcer area and opening of new ulcer).

Figure 4 shows a participant with four ulcers with favorable evolution of the healing process whereas Figure 5 depicts another participant with a single VU with unfavorable evolution despite treatment (this adverse event was considered not related to the fibrin sealant). Therefore, it was concluded that the product is safe at the proposed dose and promoted healing and wound bed preparation. 

Both studies by Gatti et al. [56] and by Abbade et al. [57] were considered “academic” from the perspective of product registry of the Brazilian Health Regulatory Agency (ANVISA). In order to get the product registered and utilize it on patients in the Brazilian public health system, it must have its clinical protocol approved simultaneously by the National Commission of Ethics in Research (CONEP) and by ANVISA. Currently, this new challenge is being faced by the research group involved.

## Advances in the Diagnostic and/or Prognostic Approach Toward Venous Ulcers by Means of Proteomic Strategies

The number of studies that seek to identify candidate molecules as diagnostic and/or prognostic markers in body fluids has been increasing in the worldwide literature. The goal of these works is based on the fact that such molecules can indicate a prognosis and/or evolution of a disease for cure or therapeutic failure [58].

Proteins are among the main molecular targets being investigated. In general, these are macromolecules responsible for controlling the majority of cellular processes acting as enzymes, antibodies, hormones, structural components or cellular receptors [59]. In this context, the investigative approach known as proteomic analysis has been broadly applied in basic research studies to identify molecular markers in sick and healthy individuals [60]. A specific or unspecific molecular marker can be evaluated quantitatively to determine the progression and/or regression of a particular disease [61]. The proteomic approach has become an important tool in daily medical practice aiming at the discovery of new drugs as well as contributing to the development of diagnostic tests [58, 62].

In the scope of VUs, the detailed knowledge of proteins produced in inflammatory exudates is a longstanding concern of researchers. It is known that an exudate has a complex composition containing among other substances, soluble factors released into the ulcer microenvironment, which in turn may influence local cellular functions. Thus, an inflammatory exudate is a liquid biopsy that reflects the metabolic condition of the wound and is therefore useful in identifying the factors involved or not involved in tissue repair [58, 63, 64, 65].

In 2008, Fernandez et al. [58] analyzed the inflammatory exudate of VUs for the first time. Using immunoprecipitation and mass spectrometry assays, they identified 40 proteins in the inflammatory fluid of these ulcers. These were associated with acute-phase, coagulation and complement-system proteins, as well as transport ones. Interestingly, the protein content in this inflammatory fluid was lower in comparison with other types of ulcers. This pioneering study made it possible to identify the first candidates for molecular markers that could contribute to the improvement of wound treatment and healing.

Broadbent et al. [63], in 2010, suggested a proteomic approach toward chronic ulcers in order to identify diagnostic/prognostic molecular markers related to their treatment and cure. They concluded that clinical research of chronic ulcers associated with proteomic tools could uncover strategically significant targets. In addition, they provided the first comprehensive summary of protein-centered analyses and recommended the proteins elastases, fibronectins and matrix metalloproteases (MMP) as potential biomarkers or targets for therapeutic intervention. These data were corroborated by Eming et al. [66] in the same year, who conducted a comparative study evaluating wound exudates obtained from normal healing or nonhealing ulcers. They identified elastases and metalloproteases as potential biomarkers involved in the healing process. They found 26 exclusive proteins in chronic ulcer inflammatory fluid, demonstrating that, because they are molecules with specific expression in a particular type of lesion, they may be suggestive of the healing process. Prominent among VU-expressed proteins are MMP-9-type metalloproteases, neutrophil elastase, proteinase-3, fibulin-1 and sulfate proteoglycan-2. All are related to the inflammatory response and tissue reconstitution. 

In a 2014 review study, Mannello et al. [61] described the majority of the known metabolites discovered in the last 25 years in VU exudate. Furthermore, these authors reinforced the use of proteins contained in ulcer inflammatory exudates as prognostic biomarkers or as possible targets for therapeutic approaches [61]. They concluded that proteomic studies provide the basis for future identification of biomarkers and establish conditions for ascertaining the appropriate combination of molecules that are expressed in the healing pathway. 

In a 2017 general review of the literature, Broszczak et al. [67] discussed the cellular and molecular mechanisms involved in healing, as well as the aspects in which this process fails, resulting in wound chronicity. The omic approaches provided information on biology and revealed markers of differentiation at the genomic, transcriptomic, proteomic and metabolomic levels. In addition, the review describes the set of analytical tools and approaches that were employed to capture multivariate data at each of these molecular levels. They concluded that in the future, the development and spatiotemporal analysis of wounds, together with the integration of multiple omic datasets, may provide insight into the main molecules that lead to chronicity. These biomarkers have potential for clinical diagnosis and may aid in the prognosis of each patient. 

**Figure 6.**
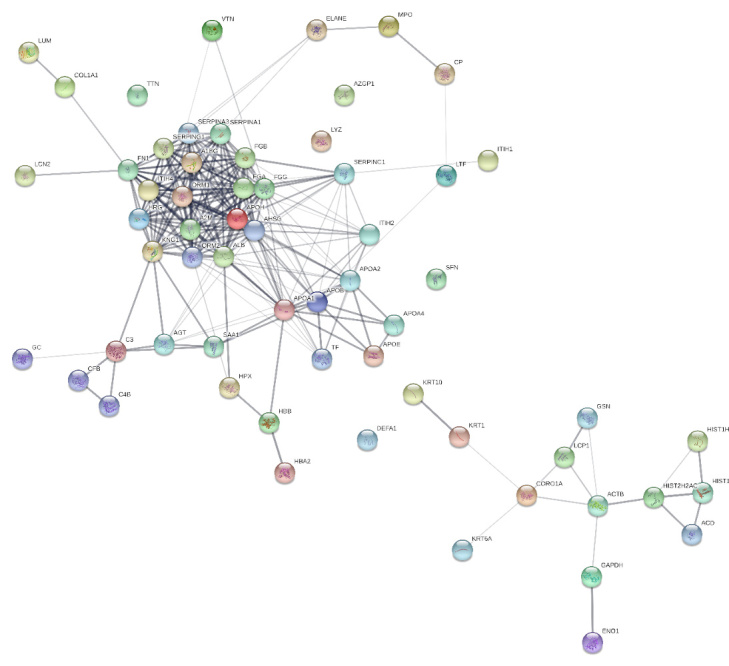
Protein-protein interactions network among all the proteins identified in the study by Cavassan et al. [68] using String software. The darker lines represent high interaction whereas lighter ones represent weaker interactions. (From: Cavassan NRV, et al. Correlation between chronic venous ulcer exudate proteins and clinical profile: A cross-sectional study. J Proteomics. 2019;192:280-90.)

In light of the importance and challenge of this subject, Cavassan et al. [68] in 2019 initiated studies in search of a correlation between the proteins present in the inflammatory exudate of 37 VUs and the clinical and epidemiological characteristics of 28 patients afflicted with these wounds. The mean age range was 70 years (± 10.1), with 62.2% being females and 37.8% males. Seventy-three percent (73%) adhered to compression and rest during the study, 81.1% reported history of primary varicose veins, 54.1% reported history of systemic arterial hypertension, 54.1% reported devitalized ulcer bed tissue and 64.9% reported ulcers with more than ten years of evolution. Using the association between the protein digestion process, mass spectrometry analysis and the use of proteomic bioinformatics tools, 76 proteins related to the healing process were identified. These authors evidenced the associations among: the disease diabetes mellitus and the presence of the proteins Ig gamma-2, apolipoprotein-A1 and albumin; congestive cardiac failure and the presence of the protein Ig lambda-2; colonization of the ulcer by microorganisms and the presence of the protein actin; compressive therapy and the presence of the protein Ig kappa; systemic arterial hypertension and the presence of the proteins alpha-2-macroglobulin and apolipoprotein-A1; ulcer area and the presence of the protein apolipoprotein-A1; race and the presence of the proteins heavy-chain Ig and Ig gamma -1 chain; age and race and the presence of Ig gamma-1 chain. The protein-protein interaction is displayed in Figure 6 [68]. Such results may be the basis for further research on diagnostic/prognostic support for predicting the chronicity of VUs.

In another work, Cavassan et al. [69] identified differentially expressed proteins in the inflammatory exudate of VUs and correlated them with the ease or difficulty of healing. The same 37 ulcers indicated in the 2019 study [68] were monitored for 90 days. All patients received standard treatment for VUs, i.e., compressive therapy and dressings according to ulcer conditions. Inflammatory exudates were analyzed at time zero (T = 0) using the Shotgun label-free strategy. At the end of 90 days, it was observed that 67.6% of the wounds presented reduction and/or healing and 32.4% non-healing or augmentation of the lesion. The authors demonstrated that C3 complement and ceruloplasmin proteins presented a higher expression in ulcers that had decreased area and/or healing, whereas apoliprotein A1 and neutrophil defensin-1 proteins showed higher expression in non-healing ulcers that either increased or did not diminish their area over the 90 days. It was then observed that the complement proteins C3, ceruloplasmin, apoliprotein a1, and neutrophil defensin-1 act as candidates for prognostic markers of healing in VUs. Multicenter studies should be conducted to validate or refute these findings. If they are conclusive, it will be possible to develop prognostic kits for VUs and anticipate the possible evolution of the disease, either by cure or permanent chronicity.

## Conclusions

Fibrin sealants are validated as scaffold and drug delivery systems [37, 70] that display excellent healing properties, thereby contributing to the treatment of VUs. The only ones available commercially are homologous ones extracted from human blood pool, which are extremely expensive but very efficient. 

This review shows the difficulty faced by health professionals worldwide who treat VUs in prescribing sealants due to their high costs. The heterologous sealant - the only one produced, developed and studied in the world until the present moment - remains non-commercial, its cost is low and it is easily produced due to the abundance of the raw material. The two clinical trials already carried out in phase I/II evidenced the safety and efficacy of the product, as well as its objective of treating VUs at the proposed doses. 

Furthermore, the analyses of liquid biopsies from VUs, utilizing the tools of proteomic strategies, have shown the possibility of predicting prior to treatment initiation which ulcers would be easy or difficult to heal. This prognosis is only possible by evaluating the expression of the proteins isolated in the exudates by means of shotgun label-free strategy. Those easily healed express mainly complement C3 and ceruloplasmin, whereas those that are difficult to heal express apoliproteína a1 and neutrophil defensina-1 [69]. 

Finally, heterologous fibrin sealant is a low-cost biopolymer that is easy to apply topically, promotes healing and contributes to wound bed preparation, especially in difficult-to-heal VUs. 

### Abbreviations

ANVISA: Brazilian Health Regulatory Agency; CONEP: National Commission of Ethics in Research; FDA: US Food and Drug Administration; HFS: heterologous fibrin sealant; MMP: matrix metalloproteases; VUs: venous ulcers.
